# Disparities in Physician Compensation by Gender in Ontario, Canada

**DOI:** 10.1001/jamanetworkopen.2021.26107

**Published:** 2021-09-21

**Authors:** Mitch Steffler, Nadine Chami, Samantha Hill, Gail Beck, Stephen C. Cooper, Robert Dinniwell, Sarah Newbery, Sarah Simkin, Brittany Chang-Kit, James G. Wright, Jasmin Kantarevic, Sharada Weir

**Affiliations:** 1 Ontario Medical Association, Toronto, Canada; 2 Canadian Centre for Health Economics, University of Toronto, Toronto, Canada; 3 Division of Cardiac Surgery, St Michael’s Hospital, Toronto, Canada; 4 Division of Cardiac Surgery, Sunnybrook Health Sciences Centre, Toronto, Canada; 5 Department of Psychiatry, Division Of Child Psychiatry, University of Ottawa, Ottawa, Canada; 6 Little Current Medical Associates, Little Current, Canada; 7 Northern Ontario School of Medicine, Sudbury, Canada; 8 Department of Radiation Oncology, Princess Margaret Hospital, University of Toronto, Toronto, Canada; 9 Department of Oncology, Schulich School of Medicine and Dentistry, Western University, London, Canada; 10 North of Superior Healthcare Group, Marathon, Canada; 11 Canadian Health Workforce Network, Ottawa, Canada; 12 Department of Family Medicine, University of Ottawa, Ottawa, Canada; 13 Temerty Faculty of Medicine, University of Toronto, Toronto, Canada; 14 Department of Surgery, University of Toronto, Toronto, Canada; 15 Institute of Health Policy, Management and Evaluation, University of Toronto, Toronto, Canada; 16 Deparment of Economics, University of Toronto, Toronto, Canada; 17 Insitute of Labour Economics, Deutsche Post Foundation, Bonn, Germany

## Abstract

**Importance:**

Men and women should earn equal pay for equal work. An examination of the magnitude of pay disparities could inform strategies for remediation.

**Objective:**

To examine gender-based differences in pay within a large, comprehensive physician population practicing within a variety of payment systems.

**Design, Setting, and Participants:**

This cross-sectional study used data from the Ontario Health Insurance Plan (OHIP) in the 2017 to 2018 fiscal year to estimate differences in gross payments between men and women physicians in Ontario, Canada. Pay gaps were calculated annually and daily. Regression analyses were used to control for observable practice characteristics that could account for individual differences in daily pay. In Canada’s largest province, Ontario, medical services are predominantly provided by self-employed physicians who bill the province’s single payer, OHIP. All physicians who submitted claims to OHIP were included. Data were analyzed from January 2020 to July 2021.

**Exposures:**

Physician gender, obtained from the OHIP Corporate Provider Database. Gender is recorded as male or female.

**Main Outcomes and Measures:**

Gross clinical payments were tabulated for individual physicians on a daily and annual basis in conjunction with each physician’s practice characteristics, setting, and specialty.

**Results:**

A total of 31 481 physicians were included in the study sample (12 604 [40.0%] women; 18 877 [60.0%] men; mean [SD] time since graduation, 23.3 [13.6] years), representing 99% of active physicians in Ontario. The unadjusted differences in clinical payments between male and female physicians were 32.8% (95% CI, 30.8%-34.6%) annually and 22.5% (95% CI, 21.2%-23.8%) daily. After accounting for practice characteristics, region, and specialty, the overall daily payment gap was 13.5% (95% CI, 12.3%-14.8%). The pay gap persisted with differing magnitudes when examined by specialty (ranging from 6.6% to 37.6%), practice setting (8.3% to 17.2%), payment model (13.4% to 22.8% for family medicine; 8.0% to 11.6% for other specialties), and rurality (8.0% to 16.5%).

**Conclusions and Relevance:**

This cross-sectional study examined differences in magnitude of annual and daily payment gaps and between unadjusted and adjusted gaps. Comparing the gaps for different specialties, geography, and payment systems illustrated the complexity of the issue by showing that the pay gap varied for physicians in different practice settings. As such, multiple directed interventions will be necessary to ensure that all physicians are paid equally for equal work, regardless of gender.

## Key Points

**Question:**

Do male and female physicians in Ontario, Canada, receive equal payments for equal work?

**Findings:**

In this cross-sectional study including 31 481 physicians (nearly all physicians in Ontario), women earned 32.8% less than men annually and 22.5% less daily. After controlling for observable factors associated with earnings, such as practice characteristics, geography (degree of rurality), and specialty, the daily pay gap remained significant, at 13.5%.

**Meaning:**

This cross-sectional study describes a complex set of factors associated with payment disparities affecting female physicians, considering both daily and annual payment gaps and the variations by specialty, rurality, and practice settings.

## Teaser

This cross-sectional study examines differences in pay by gender among physicians in Ontario, Canada.

## Introduction

As a principle of fairness, men and women should earn equal pay for equal work. This presents a challenge for the medical profession, as evidence worldwide indicates that gender pay disparities in medicine are pervasive. An annual Medscape survey of full-time US physician salaries recently reported raw gender pay gaps of 20% for family physicians (FPs) and 24% for other specialties.^1^ In dollar terms, gap estimates controlling for productive factors, such as hours worked and years of experience, have been reported ranging from US $12 000 to US $76 000 yearly.^2,3,4,5^ Gender-based pay gaps have been reported in multiple studies reflecting a variety of health systems and payment frameworks worldwide, with estimates ranging from 7% to 32%.^6,7,8,9,10,11^

In Ontario, Canada, FPs are compensated by a variety of models, including fee-for-service (FFS), capitation, and Alternative Payment Plans (APPs), while approximately two-thirds of physicians in other specialties receive FFS payments and one-third are remunerated via APPs. The fee schedule itself is blind to physician gender and other personal physician characteristics; the fee listed for each specific service applies to all physicians. However, there is evidence of billings disparities between male and female physicians in Canada. A study by Dossa and colleagues^12^ examined billing data for surgeons performing a set of common operative procedures from 2014 to 2016 and reported an hourly earnings gap of 24%, which was reduced to 14% after adjusting for surgical specialty. A study by Buys and colleagues^13^ found an unadjusted 40% gap in median annual FFS billings in Ontario over the period from 1992 through 2013. After controlling for available productive factors, the gap was 23%. These studies have established that gender disparities in physician earnings are present even in the FFS payment system, which many see as gender neutral. However, questions about the causes—and possible remedies—remain.

Previous studies on gender biases in physician earnings have often been limited by the quality of available data (eg, reliance on self-reported survey data). Many studies have failed to comprehensively include all physicians practicing within a single system or geographic region, have focused on narrow population subsets (eg, specialty-specific data), or have featured remuneration systems that are not common in Canada and the United States (eg, salary). Only 1 prior study,^14^ to our knowledge, has examined the difference and magnitude between annual and daily earnings that is critical to understanding the potential impact of different strategies for remediation.

The purpose of this study was to explore gender pay gaps in medicine for a comprehensive population of physicians using administrative health care data. Having access to all public payments made through a single-payer, universal health care system allowed for an almost complete accounting of the gender pay gap in the medical profession. Our study addresses the question of whether there are significant differences in pay between male and female physicians in Ontario and whether the magnitude of the pay gap varies based on practice characteristics, such as rurality, payment model, and specialty.

## Methods

For this cross-sectional study, formal ethics approval and informed consent were not required because we used deidentified administrative health care data that were obtained from the Ontario Ministry of Health under an agreement with the Ontario Medical Association and the research was initially carried out as part of Ontario Medical Association business operations. This study examined individual-level, publicly financed physician payments. Annual and daily payment gaps were estimated, and a multivariable regression framework was used to adjust for individual factors that could explain observed differences in payments. This study adheres to the Strengthening the Reporting of Observational Studies in Epidemiology (STROBE) reporting guideline for cross-sectional studies.

### Data Sources

Publicly funded health care in Ontario, Canada, is financed by a single payer, the Ontario Health Insurance Plan (OHIP). Clinical payment data were obtained from OHIP. Physician characteristics were taken from the Corporate Provider Database. OHIP billings (ie, FFS claims) and shadow billings (ie, records of visits and services in non-FFS models) records provided additional information on practice characteristics.

### Study Period and Population

The study population included all physicians who submitted OHIP claims, including shadow billings, from April 1, 2017, to March 31, 2018. This encompassed almost all of the practicing physician population in Ontario.

### Calculating Physician Payments

There are 3 main types of payment models in Ontario: FFS, blended capitation, and APPs. FPs in enhanced-FFS models receive 100% FFS payment plus bonuses and pay-for-performance incentives for rostered patients. Physicians in blended-capitation receive age- and gender-adjusted payments, bonuses and incentives for rostered patients, plus FFS payments for nonrostered patients.^15^ APPs include payment for academic responsibilities, emergency department services, or compensation for working in rural or isolated regions.^16^

Publicly financed payments from all sources attributable to individual physicians were aggregated at the physician level. For group-level payments, rules were used to ascribe payments to individuals. In capitation, payments were attributed based on roster size and shadow-billed amounts. Other group and APP contract amounts were split among group members based on each physician’s proportion of total billings. Data were not available on private payments for services not covered by OHIP.

Physician payments were summarized on an annual basis for the 2017 to 2018 fiscal year. Based on the number of days worked (number of days with ≥1 service billed or shadow billed), a mean daily payment amount (including FFS, capitation, and APP amounts) was computed for each physician in the study.

### Calculating Gender Pay Gaps

Physician gender was obtained from the OHIP Corporate Provider Database. Gender is recorded in OHIP as male or female. The gender pay gap was expressed as the difference between male (*M*) and female (*F*) mean payments divided by mean male payments (or equivalently, 1 − the ratio of female to male payments), expressed as:


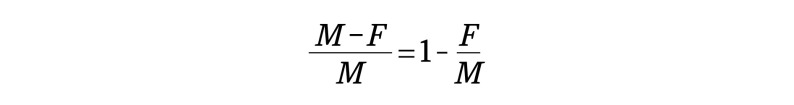


### Key Variables

The primary variable of interest was mean daily payments. This was the smallest unit of analysis available to evaluate equal pay for equal work. We also examined annual payments, which reflect the combination of mean daily payments and days worked annually.

Several covariates were included to help account for differences in male and female daily payments. Specialty was obtained from OHIP claims data. Physicians with payments in multiple specialties were assigned their dominant specialty, by dollar value. Tenure was calculated as years since graduation from medical school. Part-time work was defined as working a mean of fewer than 3 days per week (with a special exception for emergency medicine). Days with billings on weekends, holidays, and with after-hours codes recorded were each tallied as a percentage of total days worked. Academic physicians were defined as those who received any payment from an academic center in fiscal year 2017 to 2018. A physician’s institutional setting was classified as primarily hospital, private clinic, or a mix. Rurality Index of Ontario scores attached to postal code determined whether practice location was major urban (0), semiurban (1-39), or rural (≥40).^17^

### Statistical Analysis

Mean values of key outcome variables and covariates were calculated by physician gender. Bivariable (unadjusted) regression of annual or daily payments on gender was used to estimate raw gaps in annual and daily payments between male and female physicians without controlling for covariates. Multivariable regression (adjusted) models of daily payments included the following sets of explanatory variables: work inputs, degree of rurality, and practice characteristics, including specialty. Adjusted differences in daily payments among male and female physicians were also estimated separately by specialty, payment model, practice setting, and rurality to illustrate variations in the unexplained portion of the payment gap for physicians in different practice settings or situations.

All analyses were conducted using Stata version 15 (StataCorp) and SAS statistical software version 9.4 for Windows (SAS Institute). *P* values were 2-sided, and statistical significance was set at α = .05. Data were analyzed from January 2020 to July 2021.

## Results

The study population included 31 481 physicians (12 604 [40.0%] women; 18 877 [60.0%] men; mean [SD] time since graduation, 23.3 [13.6] years), representing 99% of active physicians in Ontario. Approximately 1% of physicians were excluded for 1 of the following reasons: missing record of gender or postal code, lacking individual payment information, or having anomalous payments data (ie, figures totaling ≤$0) (eTable 1 in the Supplement).

Significant differences in working characteristics were evident between male and female physicians in Ontario (Table 1). Male physicians had a mean (SD) of 26.1 (13.0) years since graduation, compared with 19.2 (11.8) years among female physicians, a difference of approximately 7 additional years of work experience, and were less likely to work part-time (4463 men [23.6%] vs 4281 women [34.0%]). Women were more likely to practice family medicine (6202 women [49.2%] vs 6940 men [36.8%]), and a smaller proportion were remunerated on a purely FFS basis (6040 women [47.9%] vs 10 327 men [54.7%]).

**Table 1.** Descriptive Statistics on Ontario Physicians, by Gender

Characteristic	No. (%)		*P* value
	Women (n = 12 604)	Men (n = 18 877)	
Clinical payments, mean (SD), $			
Annual gross clinical payments	268 044 (206 569)	402 331 (320 349)	<.001^a^
Daily gross clinical payments	1447 (935)	1870 (1255)	<.001^a^
Working characteristics			
Tenure, mean (SD), y since graduation	19.2 (11.8)	26.1 (13.0)	<.001^a^
Days worked annually, mean (SD)	176.3 (76.9)	201.6 (81.7)	<.001^a^
Days worked, mean (SD), %			
On weekends	10.5 (11.0)	12.9 (11.6))	<.001^a^
After hours	4.2 (10.6)	6.3 (13.2)	<.001^a^
Holidays	2.0 (2.3)	2.2 (2.3)	<.001^a^
Part-time status	4281 (34.0)	4463 (23.6)	<.001^a^
Received payment from academic center	1513 (12.0)	2612 (13.8)	<.001^a^
Practice setting			
Hospital	3797 (30.1)	6469 (34.3)	<.001^b^
Office	5062 (40.2)	6194 (32.8)	
Mixed	3745 (29.7)	6214 (32.9)	
Rurality^c^			
Major urban (RIO 0)	7773 (61.7)	11 024 (58.4)	<.001^b^
Semi-urban (RIO 1-39)	4029 (32.0)	6591 (34.9)	
Rural (RIO ≥40)	486 (3.9)	757 (4.0)	
No RIO score	316 (2.5)	505 (2.7)	
Specialty			
Family Medicine	6202 (49.2)	6940 (36.8)	<.001^b^
All other specialties	6402 (50.8)	11 937 (63.2)	
Payment model			
Fee for service	6040 (47.9)	10 327 (54.7)	<.001^b^
Capitation or APP (any payments)	6564 (52.1)	8550 (45.3)	

Abbreviations: APP, Alternative Payment Plans; RIO, Rurality Index of Ontario.

^a^ Based on *t* test of descriptive differences in male and female values.

^b^ Based on Pearson χ^2^ test of independence.

^c^ Based on the RIO.

### Gender Pay Gap

**Figure 1.**
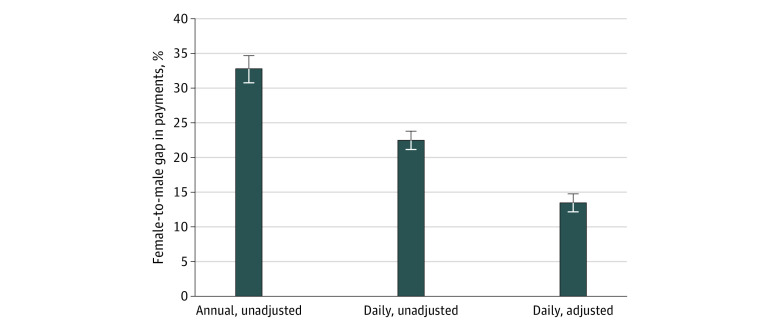
Female-to-Male Gross Clinical Payment Gap in Fiscal Year 2017 to 2018 for All Physicians Adjusted daily payment gap controls for tenure, specialty, primary care payment model, part-time status, after hours work, holiday work, weekend work, practice setting, academic physician status, and rurality. Error bars indicate 95% CIs.

The unadjusted payment gap between male and female physicians was estimated as 32.8% (95% CI, 30.8%-34.6%) annually and a mean of 22.5% (95% CI, 21.2%-23.8%) for days with billings. Men worked a mean (SD) of 201.6 (81.7) days annually, compared with 176.3 (76.9) days among women, a difference of 12.5% more days annually. Thus, approximately two-thirds of the annual pay gap was attributable to daily payments and one-third to days worked. After accounting for working characteristics, geography, and specialty (Figure 1; eTable 2 in the Supplement), the daily payment gap was reduced to 13.5% (95% CI, 12.3%-14.8%), which is equivalent to the requirement for female physicians to bill an additional 15.6% to reach the level billed by men, on an adjusted basis.

### Gender Pay Gap by Specialty

**Figure 2.**
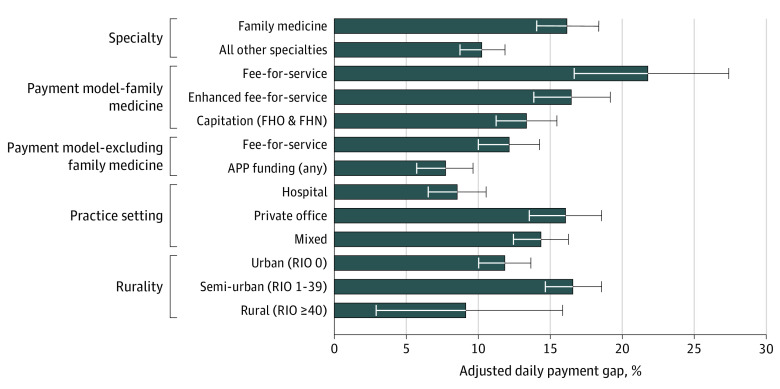
Adjusted Daily Payment Gap by Selected Practice Characteristics Adjusted daily payment gap controls for tenure, specialty, primary care payment model, part-time status, after hours work, holiday work, weekend work, practice setting, academic physician status, and rurality. APP indicates Alternative Payment Plans; FHN, Family Health Network; FHO, Family Health Organization; RIO, Rurality Index of Ontario; and error bars, 95% CIs.

Specialty accounted for a substantial portion of the daily gap. Controlling for specialty alone reduced the gap from 22.5% to 16.8% (95% CI, 15.6%-18.1%), or about two-thirds of the explained portion of the gap (eTable 2 in the Supplement). To better understand the gender pay gap among groups of physicians in Ontario, pay gaps were estimated separately by specialty. For 20 out of 36 OHIP specialties studied, adjusted differences in physician payments were statistically significant. Of these, 13 had estimated adjusted daily payment gaps of more than 15% (Table 2). Of note, the adjusted daily pay gap for FPs was 16.8% (95% CI, 14.6%-18.9%) vs 10.1% (95% CI, 8.6%-11.6%) across all other specialties (Figure 2; eTable 3 in the Supplement).

**Table 2.** Payment Gap by Specialty for the 2017 to 2018 Fiscal Year

Specialty	Physicians, No.	No. (%)		Payment gap^a^					
		Women	Men	Annual unadjusted, % (95% CI)	*P* value	Daily unadjusted, % (95% CI)	*P* value	Daily adjusted, % (95% CI)^b^	*P* value
Anaesthesia	1394	433 (31.1)	961 (68.9)	24.1 (16.1 to 31.3)	<.001	9.0 (5.0 to 12.8)	<.001	6.6 (3.0 to 10.1)	<.001
Cardiology	645	119 (18.4)	526 (81.6)	42.7 (29.4 to 53.5)	<.001	33.6 (25.5 to 40.8)	<.001	26.8 (19.7 to 33.4)	<.001
Cardiovascular and thoracic surgery	96	10 (10.4)	86 (89.6)	33.1 (−11.5 to 59.9)	.12	21.7 (−1.9 to 39.8)	.07	17.1 (−0.5 to 31.7)	.06
Clinical immunology	36	15 (41.7)	21 (58.3)	8.0 (−111.6 to 60.0)	.84	17.0 (−9.8 to 37.3)	.18	21.1 (−10.4 to 43.5)	.16
Dermatology	231	109 (47.2)	122 (52.8)	18.5 (−3.6 to 35.8)	.09	14.1 (−1.7 to 27.5)	.08	20.2 (5.5 to 32.6)	.009
Diagnostic radiology	1166	328 (28.1)	838 (71.9)	18.0 (4.3 to 29.8)	.01	12.9 (5.4 to 19.9)	.001	16.7 (10.8 to 22.3)	<.001
Emergency medicine	1972	654 (33.2)	1318 (66.8)	34.1 (27.6 to 40.0)	<.001	19.0 (15.5 to 22.2)	<.001	12.5 (9.3 to 15.6)	<.001
Endocrinology	238	134 (56.3)	104 (43.7)	33.6 (19.8 to 45.0)	<.001	15.9 (5.6 to 25.0)	.003	12.9 (2.6 to 22.2)	.02
Family medicine and general practice	13 142	6202 (47.2)	6940 (52.8)	32.8 (29.8 to 35.7)	<.001	22.3 (20.0 to 24.4)	<.001	16.8 (14.6 to 18.9)	<.001
Gastroenterology	288	70 (24.3)	218 (75.7)	19.6 (0.2 to 35.2)	.05	7.3 (−3.5 to 16.9)	.18	10.0 (−0.3 to 19.2)	.06
General surgery	843	223 (26.5)	620 (73.5)	28.7 (15.1 to 40.2)	<.001	14.6 (7.2 to 21.3)	<.001	14.5 (8.9 to 19.8)	<.001
Genetics	35	26 (74.3)	9 (25.7)	−2.6 (−81.3 to 41.9)	.93	32.9 (4.6 to 52.7)	.03	−7.0 (−54.3 to 25.8)	.71
Geriatrics	145	86 (59.3)	59 (40.7)	43.2 (23.7 to 57.6)	<.001	25.8 (10.6 to 38.5)	.002	23.0 (8.4 to 35.2)	.003
Hematology	195	91 (46.7)	104 (53.3)	−3.0 (−57.1 to 32.4)	.89	11.8 (−7.6 to 27.7)	.21	19.3 (2.9 to 32.9)	.02
Infectious disease	161	69 (42.9)	92 (57.1)	43.5 (20.3 to 60.0)	.001	11.7 (−4.4 to 25.2)	.14	3.6 (−15.2 to 19.3)	.68
Internal medicine	1829	564 (30.8)	1265 (69.2)	17.0 (5.4 to 27.2)	.005	5.8 (−0.1 to 11.4)	.05	5.5 (0.1 to 10.7)	.05
Medical oncology	218	101 (46.3)	117 (53.7)	−23.2 (−56.6 to 3.2)	.09	−6.1 (−21.4 to 7.2)	.38	−2.1 (−15.7 to 9.9)	.75
Nephrology	183	54 (29.5)	129 (70.5)	39.0 (20.9 to 53.0)	<.001	24.9 (12.6 to 35.5)	<.001	18.4 (6.3 to 29.0)	.004
Neurology	439	151 (34.4)	288 (65.6)	19.1 (0.8 to 34.0)	.04	8.3 (−1.4 to 17.0)	.09	5.0 (−4.9 to 14.0)	.31
Neurosurgery	109	10 (9.2)	99 (90.8)	58.4 (6.2 to 81.5)	.04	40.7 (8.2 to 61.7)	.02	37.6 (14.6 to 54.4)	.004
Nuclear medicine	54	10 (18.5)	44 (81.5)	−42.6 (−238.7 to 40)	.41	−9.7 (−68.0 to 28.4)	.67	11.5 (−7.5 to 27.2)	.21
Obstetrics and gynecology	949	547 (57.6)	402 (42.4)	6.5 (−8.2 to 19.3)	.36	−0.9 (−8.5 to 6.2)	.81	5.4 (−0.8 to 11.2)	.09
Ophthalmology	488	107 (21.9)	381 (78.1)	26.2 (1.5 to 44.7)	.04	15.9 (0.8 to 28.7)	.04	19.4 (6.8 to 30.3)	.004
Orthopedic surgery	655	57 (8.7)	598 (91.3)	50.7 (33.6 to 63.4)	<.001	27.4 (17.2 to 36.4)	<.001	15.9 (8.8 to 22.5)	<.001
Otolaryngology	298	56 (18.8)	242 (81.2)	14.0 (−23.4 to 40.1)	.41	6.2 (−15.9 to 24.1)	.55	10.4 (−5.1 to 23.7)	.18
Pediatrics	1555	889 (57.2)	666 (42.8)	24.9 (16.6 to 32.3)	<.001	8.6 (3.4 to 13.5)	.001	10.1 (4.5 to 15.4)	.001
Pathology	314	113 (36)	201 (64)	35.9 (−7.3 to 61.7)	.09	18.9 (−4.3 to 37.0)	.10	−0.5 (−18.8 to 15.0)	.95
Physical medicine	213	75 (35.2)	138 (64.8)	8.8 (−24.3 to 33.1)	.56	21.3 (7.0 to 33.4)	.005	21.3 (8.3 to 32.5)	.002
Plastic surgery	246	66 (26.8)	180 (73.2)	−17.7 (−73.1 to 20.0)	.41	−10.6 (−35.8 to 10.0)	.34	−5.7 (−20.6 to 7.4)	.41
Psychiatry	2202	931 (42.3)	1271 (57.7)	17.0 (8.4 to 24.8)	<.001	5.6 (1.1 to 9.9)	.02	−0.4 (−4.0 to 4.6)	.86
Respiratory diseases	297	101 (34)	196 (66)	25.7 (6.7 to 40.8)	.01	17.7 (8.5 to 26.1)	<.001	14.0 (5.6 to 21.6)	.002
Rheumatology	208	108 (51.9)	100 (48.1)	24.7 (5.5 to 39.9)	.01	19.8 (9.9 to 28.6)	<.001	22.9 (12.7 to 32.0)	<.001
Therapeutic radiology	216	60 (27.8)	156 (72.2)	11.7 (−12.1 to 30.5)	.31	10.5 (0.9 to 19.2)	.03	10.8 (−0.5 to 20.8)	.06
Thoracic surgery	44	7 (15.9)	37 (84.1)	−4.0 (−77.3 to 39.0)	.88	7.3 (−37.8 to 37.6)	.70	15.5 (−5.7 to 32.5)	.14
Urology	303	19 (6.3)	284 (93.7)	36.4 (−3.1 to 60.8)	.07	15.5 (−5.7 to 32.5)	.14	11.0 (−3.8 to 23.7)	.14
Vascular surgery	68	6 (8.8)	62 (91.2)	24.7 (−28.6 to 55.9)	.29	27.9 (−3.3 to 49.8)	.07	26.2 (2.4 to 44.2)	.03

^a^ Payment gap is expressed as 1 minus the ratio of female to male billings. Exponentiated coefficients are reported alongside associated *P* values from *t* tests on regression coefficients. Findings are based on gross clinical payments in the 2017 to 2018 fiscal year and do not consider overhead costs or earnings from nonpublic sources.

^b^ Adjusted for tenure, part-time status, after hours work, holiday work, weekend work, primary care payment model, practice setting, academic physician status, and rurality.

### Gender Pay Gap in Different Settings

For FPs and other specialties, there were differences in the gap by payment model (Figure 2). The adjusted daily payment gap for FPs was largest among physicians practicing in FFS (22.8%; 95% CI, 17.3%-28.0%) and smallest among those practicing within capitation (13.4%; 95% CI, 11.3%-15.5%). Among FPs in enhanced FFS, the adjusted daily payment gap was 17.1% (95% CI, 14.4%-19.7%). Among other specialties with any APP payments, the adjusted daily payment gap was estimated to be 8.0% (95% CI, 6.1%-10.0%), compared with 11.6% (95% CI, 9.5%-13.7%) among physicians who billed purely FFS.

The payment gap also varied across practice settings. For physicians who practiced most frequently in a hospital setting, the gap was 8.3% (95% CI, 6.3%-10.3%) after adjustments, which was almost half that found among physicians who practiced primarily in private offices (17.2%; 95% CI, 14.7%-19.6%). The adjusted gap for those who practiced in a mix of hospital and office settings was 14.6% (95% CI, 12.7%-16.5%). Excluding family medicine, a similar, if less pronounced, pattern emerged (eTable 3 in the Supplement).

Variation was also found by geographic setting. In adjusted terms, an 8.0% (95% CI, 1.3%-14.3%) payment gap was found for rural settings compared with 16.5% (95% CI, 14.6%-18.4%) in semi-urban settings and 12.1% (95% CI, 10.4%-13.9%) in urban settings.

## Discussion

This cross-sectional study provided evidence on the gender pay gap in gross physician billings and clinical payments, which account for most physician income. Estimates of annual and daily payment gaps were made across specialty and payment model for a comprehensive population of physicians in Ontario. The substantial, unexplained payment differentials have implications for equity. While our overall findings were broadly aligned with prior research from the US and internationally,^2,3,4,5,6,7,8,9,10,11^ Ontario’s rich administrative databases offered unique perspectives on the topic.

First, the annual payment gap was larger than the daily gap, reflecting the impact of gender-based differences in the number of days worked on annual payments. More research is needed to understand barriers that prevent female physicians from working as many days annually as men. Differences may arise as a result of women assuming a disproportionate share of family labor, considering prior research for Ontario by Wang and Sweetman^18^ found no gender difference in physician labor supply after controlling for parental status. Improving opportunities for organizing work around family responsibilities and policies to encourage male participation in home responsibilities (eg, parental leave) may help to alleviate this inequity. However, changing societal expectations around gender is a complex topic, and a more in-depth discussion falls outside of the scope of this study.

Second, differences between unadjusted and adjusted daily payments suggest that specialty and other practice characteristics explain a portion of the gap. However, mediating factors may themselves be confounded by intrinsic gender bias. For example, a 2020 Canadian study by Cohen and Kiran^19^ found that men tend to be concentrated in higher paying specialties. In our study, statistically significant pay gaps were found both in relatively lower-paying specialties that are dominated by female physicians, such as pediatrics and geriatrics, and in higher-paying specialties in which female physicians have just begun to make inroads, such as neurosurgery and orthopedic surgery. Thus, overcoming barriers to entry, while important in its own right, cannot be relied on to eliminate the pay gap.

Adjusted daily payment gaps were also found to vary by payment model, practice setting, and rurality. These findings indicate the complexity of the pay gap issue and suggest that different causes may exist for physicians in different practice situations. The smaller gap among physicians who received any APP or capitation payments compared with those who billed purely FFS supports the notion that innovative payment models could be a vehicle to improve pay equity. Although our study did not provide information on the causes of the gap between APP or capitation and FFS, FFS has been shown in prior research to incentivize productivity.^20^ Alternative payment schemes that incentivize efficiency or quality may offer options tailored to practice styles favored by recent graduates, many more of whom are women. However, it is not clear to what extent results may be influenced by self-selection into various payment models^21,22^ or by unobserved redistribution of group payments; more transparent funding structures would obviate the need to make such assumptions. Smaller payment gaps among hospital-based physicians and those in rural environments may partly be related to how physician work is structured in these settings, including differences in physician referral networks and practices.

Our findings suggest that no single factor fully explains the gender pay gap, and this implies that no single intervention is likely to fully address it. For example, comparing annual vs daily payment gaps suggests the need to examine policies to financially support parents and caregivers of all genders and assess the impact of these policies on the pay gap. Similarly, examining unadjusted and adjusted daily payments, and exploring differences by payment model and specialty, suggest the need to develop and implement policies to address unjustified differences in pay across and within specialties, likely via modernizing the province’s fee schedule and payment models. Our findings by rurality and practice setting suggest that referral opportunities may play a role in the pay gap. Additional research on the association between referrals and gender differences in pay could help inform the value of developing a gender-blinded referrals system. Furthermore, our findings suggest that offering individual physicians more opportunity to choose payment models and practice settings that best suit their practice style is a matter of gender equity and fairness.

There is a common misperception that a gender pay gap cannot exist in FFS health care because the fee schedule is gender blind. In this view, differences in male and female pay reflect women’s preferences regarding specialty and effort, choices they are assumed to make freely. It is important to note that determinants of income often ascribed to personal choice may actually reflect gender discrimination. For example, there is a perception that female physicians earn less because they choose to work less than their male counterparts. However, recent US-based research found that although female physicians had lower revenue from primary care visits compared with male physicians as a result of fewer patient visits, they spent more time with their patients per visit on a daily and annual basis.^14^ Reforming payments to reflect time spent with patients in FFS and to remunerate based on clinical complexity in capitated settings might go some way toward addressing gender pay gaps. The expectations placed on physicians by patients, colleagues, and society at large may be vastly different according to a physician’s gender. The impact this has on one’s work is difficult to measure. These examples highlight the need to better understand causes of the pay gap and identify targeted policies to remedy disparities.

### Limitations

This study has some limitations. This study’s reliance on administrative payment data is subject to inherent limitations. First, the smallest time unit available in the data was number of days worked. The number of hours worked would have allowed for more precise comparisons, as one might posit that men and women routinely work days of different lengths. However, an Ontario study using hours of work for specific procedures still found gaps of comparable magnitude with those reported here.^12^ Second, we were unable to account for overhead costs, which consume a substantial portion of physician revenues and vary by specialty.^23^ There is little prior research to guide assumptions about whether overhead costs vary by physician gender. However, it is plausible that a net pay gap could be even greater. Third, some payment data were unavailable. Salaried physicians, like those in nonclinical roles (eg, hospital administration), were not included in the study. In addition, the relatively small contribution of private payments for non-OHIP services could not be studied. Fourth, we made assumptions about how group-level payments were distributed to individual physicians in APP and capitation models. Allocating payments either by patient roster or shadow billings ignores the possibility of an internal redistribution of payments at the group level. Without detailed knowledge of group contracts, the direction and magnitude of this potential bias is unclear. Fifth, we lacked data on nonbinary gender, transgender status, sexual orientation, race/ethnicity, and other factors that may intersect with gender and magnify gaps.

## Conclusion

This cross-sectional study found significant differences in payments between men and women, even after controlling for physician and practice characteristics. We found differences in the magnitude of the gender gap across specialty, payment model, geography, and practice setting. As jurisdictions around the world look to prioritize pay equity in fee setting and contract negotiations, these findings are important for identifying and designing the best policies to support these aims.

## Notes

Supplement. **eTable 1.** Study Population **eTable 2.** OLS Regression Results on Natural Log of Billings for All Physicians **eTable 3.** Female-to-Male Payment Gaps by Category
