# Supplementary material for: Disparities in Physician Compensation by Gender in Ontario, Canada
Source: JAMA Netw Open. 2021 Sep 21;4(9):e2126107. doi: 10.1001/jamanetworkopen.2021.26107 (PMC8456385; doi:10.1001/jamanetworkopen.2021.26107)
Supplement: Supplement. — eTable 1. Study Population eTable 2. OLS Regression Results on Natural Log of Billings for All Physicians eTable 3. Female-to-Male Payment Gaps by Category [file jamanetwopen-e2126107-s001.pdf]

Supplemental Online Content

Steffler M, Chami N, Hill S, et al. Disparities in physician compensation by gender in Ontario, Canada. *JAMA Netw Open*. 2021;4(9):e2126107. doi:10.1001/jamanetworkopen.2021.26107

eTable 1. Study Population

eTable 2. OLS Regression Results on Natural Log of Billings for All Physicians

eTable 3. Female-to-Male Payment Gaps by Category

This supplemental material has been provided by the authors to give readers additional information about their work.

© 2021 Steffler M et al. *JAMA Network Open.*

eTable 1. Study Population

**Inclusion/ Exclusion Criteria**	**Number Removed**	**Total Remaining**	**% of FY 2017 Billers Removed**
Any physician with billing records in FY 2017	-	31,798	-
Exclusions:			
Missing Record of "Sex" on File	19	31,779	0.1%
Missing or Invalid Ontario Postal Code	9	31,770	0.0%
*Missing Individual Income Information**	253	31,517	0.8%
Anomalous Billing Information (Billings total ≤$0)	36	31,481	0.1%
Final Study Population	-	31,481	1.0%

*Fees from Telehealth, Laboratory Medicine, and Community Palliative on-call codes were excluded from the analysis. Cases where billings could not reasonably be attributed to individuals were also excluded.

© 2021 Steffler M et al. *JAMA Network Open.*

eTable 2. OLS Regression Results on Natural Log of Billings[a,b] for All Physicians

		Annual Unadjusted (95% CI)	*P* value	Daily Unadjusted (95% CI)	*P* value	Daily Within Specialty[c] (95% CI)	*P* value	Daily Adjusted[c] (95% CI)	*P* value
**Physician Sex**	Female<br>ref: Male	0.672 (0.654-0.692)	p<0.001	0.775 (0.762-0.788)	p<0.001	0.832 (0.819-0.844)	p<0.001	0.865 (0.852-0.877)	p<0.001
**Working Characteristics**	Years since graduation							1.023 (1.021-1.025)	p<0.001
	Years since graduation, squared							1.000 (1.000-1.000)	p<0.001
	Part-time status (<3 days/week)							0.750 (0.735-0.765)	p<0.001
	Weekend days worked as a percentage of total days worked annually							1.005(1.004-1.006)	p<0.001
	Days with after-hours worked as a percentage of total days worked annually							1.010 (1.009-1.011)	p<0.001
	Holidays worked as a percentage of total days worked annually							1.008 (1.001-1.014)	0.02
**Practice Setting**	Mixed practice<br>ref: 95%+ Hospital based							0.932 (0.909-0.956)	p<0.001
	95%+ Private clinic<br>ref: 95%+ Hospital based							0.969 (0.952-0.986)	0.001
**Academic Status**	Received any academic funding							1.037 (1.016-1.060)	0.001
**Rurality (based on the Rurality Index of Ontario)**	Semi-urban (RIO=1-39)<br>ref: Urban (RIO=0)							1.048 (1.033-1.064)	p<0.001
	Rural (RIO=40-100)<br>ref: Urban (RIO=0)							0.852 (0.821-0.884)	p<0.001
	RIO Missing<br>ref: Urban (RIO=0)							0.924 (0.877-0.973)	0.003
**R-squared**		0.02		0.03		0.28		0.36	
**N**		31,481		31,481		31,481		31,481	

Notes:

a) Exponentiated coefficients are reported.
b) Findings are based on gross clinical payments in FY 2017 and do not consider overhead or earnings from non-public sources.
c) Daily Adjusted and Daily Within Specialty results include specialty-level fixed effects.

© 2021 Steffler M et al. *JAMA Network Open.*

eTable 3. Female-to-Male Payment Gaps[a,b] by Category

		Annual Unadjusted (95% CI)	*P* value	Daily Unadjusted (95% CI)	*P* value	Daily Adjusted[c] (95% CI)	*P* value
**Overall**		32.8% (30.8%-34.6%)	p<0.001	22.5% (21.2%-23.8%)	p<0.001	13.5% (12.3%-14.8%)	p<0.001
**Specialty**	Family Medicine	32.8% (29.8%-35.7%)	p<0.001	22.3% (20.0%-24.4%)	p<0.001	16.8% (14.6%-18.9%)	p<0.001
	All Other Specialties	29.7% (27.0%-32.3%)	p<0.001	16.9% (15.2%-18.5%)	p<0.001	10.1% (8.6%-11.6%)	p<0.001
**Family Practice Payment Model**	Fee-for-Service	42.4% (36.3%-48.0%)	p<0.001	31.8% (27.2%-36.2%)	p<0.001	22.8% (17.3%-28.0%)	p<0.001
	Enhanced FFS	28.5% (25.0%-31.8%)	p<0.001	19.1% (16.5%-21.7%)	p<0.001	17.1% (14.4%-19.7%)	p<0.001
	Capitation (FHO & FHN)	29.1% (27.2%-31.0%)	p<0.001	17.5% (15.7%-19.4%)	p<0.001	13.4% (11.3%-15.5%)	p<0.001
	Other PEM	26.5% (3.4%-44.1%)	0.03	17.4% (-1.8%-32.9%)	0.07	15.1% (-0.4%-28.2%)	0.06
**Payment Model – Excluding Family Practice**	Fee-For-Service	32.8% (28.9%-36.4%)	p<0.001	19.1% (16.8%-21.4%)	p<0.001	11.6% (9.5%-13.7%)	p<0.001
	APP Funding (Any)	27.0% (23.6%-30.2%)	p<0.001	15.3% (13.2%-17.3%)	p<0.001	8.0% (6.1%-10.0%)	p<0.001
**Practice Setting**	Hospital	26.7% (22.8%-30.4%)	p<0.001	15.9% (13.7%-18.2%)	p<0.001	8.3% (6.3%-10.3%)	p<0.001
	Private Office	28.4% (24.6%-32.1%)	p<0.001	19.8% (17.2%-22.4%)	p<0.001	17.2% (14.7%-19.6%)	p<0.001
	Mixed	39.1% (36.8%-41.4%)	p<0.001	27.3% (25.4%-29.1%)	p<0.001	14.6% (12.7%-16.5%)	p<0.001
**Practice Setting – Excluding Family Practice**	Hospital	24.3% (20.1%-28.3%)	p<0.001	13.0% (10.6%-15.3%)	p<0.001	6.9% (4.9%-8.8%)	p<0.001
	Private Office	26.0% (17.0%-34.0%)	p<0.001	15.2% (9.8%-20.2%)	p<0.001	13.8% (9.0%-18.4%)	p<0.001
	Mixed	35.4% (32.3%-38.3%)	p<0.001	21.8% (19.6%-24.0%)	p<0.001	12.7% (10.4%-14.9%)	p<0.001
**Rurality (based on the Rurality Index of Ontario)**	Urban (RIO=0)	32.5% (29.9%-35.0%)	p<0.001	21.7% (19.9%-23.5%)	p<0.001	12.1% (10.4%-13.9%)	p<0.001
	Semi-Urban (RIO=1-39)	33.6% (30.6%-36.4%)	p<0.001	23.8% (21.9%-25.7%)	p<0.001	16.5% (14.6%-18.4%)	p<0.001
	Rural (RIO=40-100)	23.6% (10.2%-35.0%)	0.001	19.2% (11.7%-26.2%)	p<0.001	8.0% (1.3%-14.3%)	0.02

Notes:

a) Payment Gap is expressed as one minus the ratio of female to male billings. Exponentiated coefficients are reported.

b) Findings are based on gross clinical payments in FY 2017 and do not consider overhead or earnings from non-public sources.

c) Daily Adjusted controls for tenure, specialty, primary care payment model, part-time status, after hours work, holiday work, weekend work, practice setting, academic physician status and rurality.

© 2021 Steffler M et al. *JAMA Network Open.*
